# Mental Health Questions on State Medical License Applications and Evaluation of Updates

**DOI:** 10.1001/jamanetworkopen.2023.33360

**Published:** 2023-09-12

**Authors:** Rachel N. Douglas, Emily E. Sharpe, Molly Kraus, Daniel Saddawi-Konefka, Andrew C. Hanson, Bridget Pulos

**Affiliations:** 1 Department of Anesthesiology and Perioperative Medicine, Mayo Clinic, Rochester, Minnesota; 2 Department of Anesthesiology and Perioperative Medicine, Mayo Clinic, Phoenix, Arizona; 3 Department of Anesthesia, Critical Care, and Pain Medicine, Massachusetts General Hospital, Boston; 4 Division of Biomedical Statistics and Informatics, Mayo Clinic, Rochester, Minnesota

## Abstract

This cross-sectional study evaluates the consistency of US medical license renewal applications with the Federation of State Medical Boards recommendations for questions regarding physician mental health.

## Introduction

Many physicians avoid mental health care, fearing it could lead to the loss of their medical license.^1,2,3^ In 2014, the US Supreme Court ruled that to be compliant with the Americans with Disabilities Act (ADA) professional licensing boards must limit mental health questions to current diagnoses impairing the applicant’s ability to perform professional duties.^4^ In 2018, the Federation of State Medical Boards (FSMB) released 4 recommendations for medical boards to be compliant with the ADA and promote physician wellness on medical license applications: (1) ask only if impaired, (2) ask only current, (3) allow for safe haven nonreporting, and (4) include supportive language normalizing physician wellness.^5,6^

The main objective of this study is to evaluate consistency with FSMB recommendations for state or territory medical license renewal applications. Secondary objectives include assessing the consistency between initial and renewal applications and examining the progress made in following FSMB guidelines for initial licensing applications since 2020. To our knowledge, this is the first study assessing the FSMB recommendation consistency on renewal applications.

## Methods

This cross-sectional study follows the Strengthening the Reporting of Observational Studies in Epidemiology (STROBE) reporting guideline. The Mayo Clinic institutional review board waived informed consent because data were publicly available.

Between March and December 2022, initial and renewal applications from 55 US state and territory allopathic medical licensing boards were collected and scored by 2 authors (R.D. and B.P.) using previously described methods.^6^ Disagreements were resolved through group consensus. The number of applications meeting each recommendation was summarized. Differences between initial and renewal applications and 2020 and 2022 initial applications^6^ were summarized with absolute differences. Because the analysis covers nearly the entire finite population, no formal hypothesis testing was warranted. Data were summarized using R version 4.1.2 (R Foundation for Statistical Computing) between February and April 2023.

## Results

Initial and renewal medical license applications were obtained from all 50 states, Washington DC, and 4 US territories. For renewal applications, only 3 states or territories (5%) met all 4 recommendations, 28 (51%) met 3 recommendations, 12 (22%) met 2 recommendations, 9 (16%) met 1 recommendation, and 3 (5%) met no recommendations (Figure).

**Figure.**
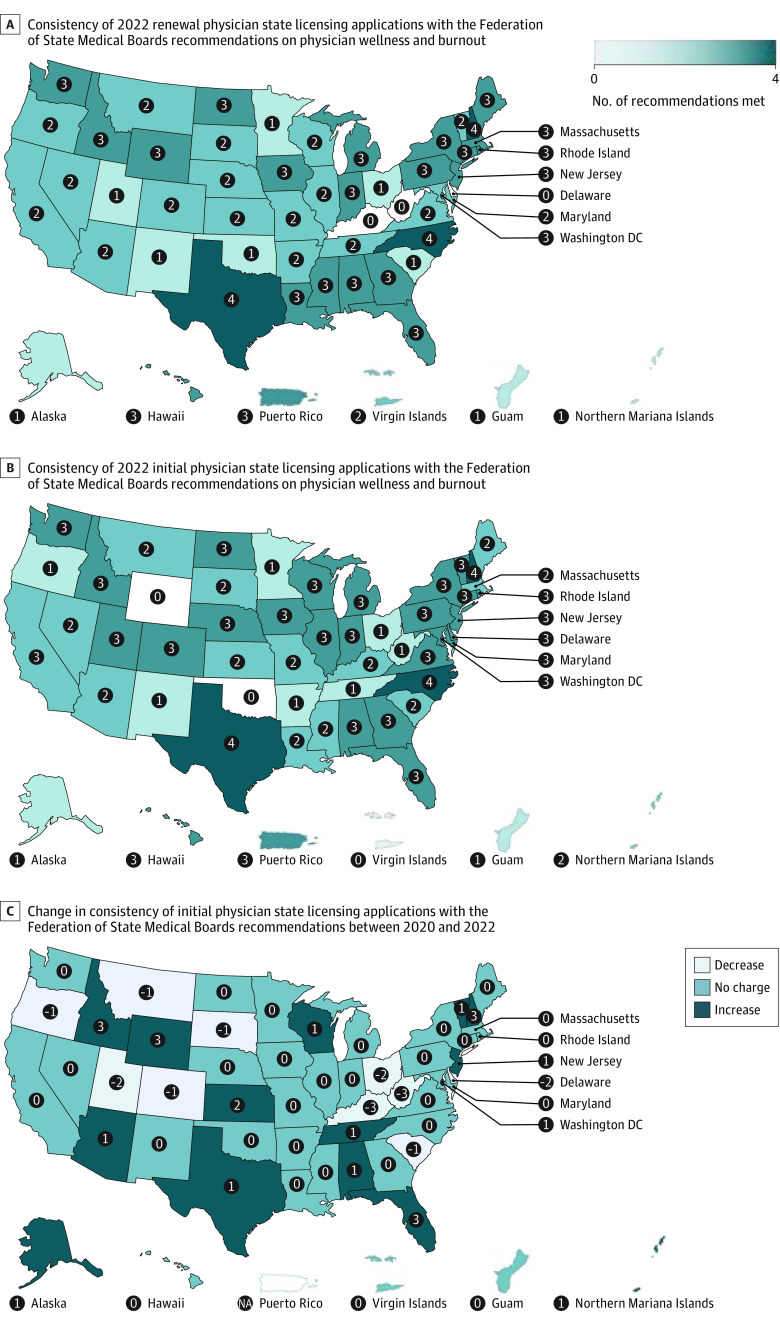
Consistency of Physician State Licensing Applications With the Federation of State Medical Boards Recommendations on Applicant Mental Health Questions NA indicates not available.

In 2022, fewer renewal applications met the only if impaired criteria compared with initial applications (40 of 55 [73%] vs 47 of 55 [85%]), but renewal applications more often met criteria for safe haven nonreporting (33 of 55 [60%] vs 23 of 55 [42%]), and only current (49 of 55 [89%] vs 46 of 55 [84%]) compared with initial applications (Figure and Table).

**Table.** Comparison of Federation of State Medical Boards Recommendations Met on Medical License Applications

Characteristics	2020 Initial (n = 54)		2022 Initial (n = 55)		2022 Renewal (n = 55)		Discordance, 2020 to 2022 initial		Discordance, 2022 initial to renewal	
	Met	Not met	Met	Not met	Met	Not met	Gain or loss/Total (%)^a^	State	Gain or loss/Total (%)^b^	State
Only if impaired	39 (72)	15 (28)	47 (85)	8 (15)	40 (73)	15 (27)	Gain: 12/15 (80); loss: 5/39 (12)	Gain: AL, AK, AZ, FL, ID, KS, NH, ND, MP, OR, TN, WY; loss: DE, KY, SC, UT, WV	Gain: 3/8 (38); loss: 10/47 (21)	Gain: DE, KY, UT; loss: AL, AK, AR, LA, ME, MT, OH, TN, USVI, WY
Only current	41 (76)	13 (24)	46 (84)	9 (16)	49 (89)	6 (11)	Gain: 8/13 (62); loss: 4/41 (9.8)	Gain: FL, ID, KS, MN, NH, VT, WI, WY; loss: DE, KY, OH, WV	Gain: 7/9 (78); loss: 4/46 (9)	Gain: AK, AZ, DE, KY, MP, OH, WV; loss: OK, OR, USVI, WY
Safe haven	25 (46)	29 (54)	23 (42)	32 (58)	33 (60)	22 (40)	Gain: 5/29 (17); loss: 8/25 (32)	Gain: DC, ID, NH, NJ, WY; loss: IA, KY, MN, OH, OR, SD, UT, WV	Gain: 12/32 (38); loss: 2/23 (9)	Gain: CA, DE, FL, IL, IA, MD, MT, NE, SC, UT, VT, VI; loss: ME, WY
Supportive language	8 (15)	46 (85)	7 (13)	48 (87)	7 (13)	48 (87)	Gain: 3/46 (7); loss: 4/8 (50)	Gain: FL, IA, TX; loss: CO, MT, ND, OR	Gain: 4/48 (8); loss: 4/7 (57)	Gain: AL, CO, ME, WI; loss: AZ, FL, IA, MA

Abbreviation: MP, Northern Mariana Islands.

^a^ Gain is the number meeting recommendations in 2022 having not met in 2020. Loss is the number not meeting recommendations in 2022 having met in 2020. Denominators and percentages are presented. One additional territory was available in 2022, and therefore, the 2020 and gain or loss counts will not sum to the 2022 counts without accounting for the additional territory. The additional territory met criteria for only if impaired, only current, and safe haven.

^b^ Gain is the number of states or territories in 2022 meeting recommendations in renewal but not initial applications. Loss is the number of states or territories not meeting recommendations in 2022 renewal but was met in the 2022 initial. Denominators and percentages are presented.

Compared with 2020 initial applications, more 2022 initial applications met the criteria for only if impaired (47 of 55 [85%] vs 39 of 54 [72%]) and only current (46 of 55 [84%] vs 41 of 54 [76%]) recommendations. However, the 2022 initial applications met safe haven nonreporting (23 of 55 [42%] vs 25 of 54 [46%]) and supportive language criteria less frequently (7 of 55 [13%] vs 8 of 54 [15%]) (Figure and Table).

## Discussion

The findings of this cross-sectional study suggest that the consistency of renewal applications with FSMB guidelines is notably low, particularly for safe haven nonreporting and supportive language. Notably, the renewal application of only 3 states or territories met all recommendations, suggesting that substantial work is needed to fully support our health care workforce in an era marked by rising physician burnout and mental health issues.

There was also marked heterogeneity between initial and renewal applications within the same state. Notably, renewal applications fared far better in safe haven nonreporting. Addressing these within-state discrepancies offers a straightforward and meaningful opportunity to support physician mental health care.

Progress since 2020 has been mixed. Initial applications demonstrated an improvement in meeting the only if impaired and only current recommendations, which may suggest an emphasis on ADA compliance. It is concerning that consistency with safe haven nonreporting and supportive language has not improved because reducing stigma is critical to removing barriers to care access. A paradigm shift is needed, where boards see supporting physician mental health as crucial to safeguarding public health.

This study had limitations. One limitation was potential changes to applications within the review period. Future research should investigate the association of safe haven nonreporting and supportive language with physicians mental health and their awareness of treatment options.

## Notes

Supplement.
Data Sharing Statement
