# Errors in Introduction

**DOI:** 10.1001/jamanetworkopen.2023.53351

**Published:** 2024-01-05

In the Original Investigation titled “Mental Health Questions on State Medical License Applications and Evaluation of Updates,”^1^ published September 12, 2023, there was an error in the wording in the second sentence of the Introduction. The sentence originally described the reference as a US Supreme Court ruling, and it should have described the reference as a US Department of Justice settlement. This article has been corrected.^1^
